# A Study of Directionality Effects in Three-Beam Coaxial Titanium Wire-Based Laser Metal Deposition

**DOI:** 10.3390/ma17133201

**Published:** 2024-06-30

**Authors:** Remy Mathenia, Braden McLain, Todd Sparks, Frank Liou

**Affiliations:** 1 Department of Mechanical and Aerospace Engineering, Missouri University of Science and Technology, Rolla, MO 65409, USA; remymathenia@mst.edu (R.M.); btmywv@mst.edu (B.M.); 2 Product Innovation and Engineering, St. James, MO 65559, USA; toddesparks@mopine.com

**Keywords:** three-beam coaxial laser, wire deposition, bead geometry, defocusing

## Abstract

Coaxial wire-based laser metal deposition is a versatile and efficient additive process that can achieve a high deposition rate in the manufacturing of complex structures. In this paper, a three-beam coaxial wire system is studied, with particular attention given to the effects of the deposition direction and laser beam orientation on the resulting bead geometry symmetry. With the three-beam laser delivery, the laser spot pattern is not always symmetric with respect to the deposition direction. Single titanium beads are deposited in different directions and at varying deposition rates, and the bead profile is quantitatively scored for multiple symmetry measures. Through an analysis of variance, the deposition direction and deposition rate were found to be insignificant with respect to the resulting bead symmetry for the developed measures. The bead symmetry and geometry are important factors in determining if a build is free of critical defects, and in this study, it is shown that the three-beam coaxial wire deposition setup is a directionally independent process.

## 1. Introduction

For the production of complex, one-of-a-kind, and large parts, the additive manufacturing (AM) strategy of directed energy deposition serves as a promising and effective alternative to traditional manufacturing methods [1]. The directed energy deposition (DED) process combines a continuous material feed and energy source to additively manufacture near-net shape components. In DED, lasers are commonly used as a concentrated energy source to melt the feedstock material. Lasers are used in many manufacturing methods as the primary energy source, such as additive manufacturing, welding, ablation, engraving, cutting, drilling, and micromachining. DED has the benefits of saving time and material waste over classical manufacturing methods and other AM strategies [2]. Wire-fed DED systems are used to achieve high deposition rates and/or large builds. Such applications are most often found in the aerospace industry as a replacement technology for large forgings and castings. Although wire systems generally have more surface roughness and a lower achievable resolution than powder-based DED, they realize the benefits of decreased waste, increased efficiency, easier material preparation, and a low-defect, high-quality material [3]. On the other hand, powder DED has the advantages of increased precision, better resolution, and more efficient energy absorption. These benefits come at the expense of a decreased material printing rate, more tedious and demanding material preparation, increased waste production, and potentially creating a health hazard with airborne metallic particulates [4]. The material delivery strategy needs to be carefully considered based on the needs and requirements of the manufactured component and the capabilities of the available technologies.

Wire-based DED generally falls into two main categories of side-feed systems and coaxial systems. In a conventional side-feed wire–laser setup, the laser is delivered to the process at an off-axis angle. This causes the process results to be dependent on the deposition direction. Conversely, coaxial wire-based deposition combats this directional dependence by feeding the wire and laser on the same axis. The wire is delivered to the process perpendicular to the deposition surface, and the laser is delivered around the wire. When comparing the side-feed and coaxial systems, the coaxial process is less sensitive to the deposition travel direction due to the laser delivery strategy [5]. Since the laser beam has to be split and carefully aimed at the wire, the optics head in a coaxial setup is generally more complex [6,7]. The laser profile is commonly annular or involves multiple discrete beams spaced around the wire. The focus of this paper will be on three-beam laser systems.

Critical deposition processes are the laser delivery style and angle, laser spot size, traverse and wire feed speeds, and laser power [8]. Other factors that are critical to a deposit are the wire diameter and material and part shape and size. The quality of the printed material is determined by the process parameter combination and temporal considerations of the deposition process. A unique feature of the laser and wire arrangement is that the standoff distance of the coaxial deposition head to the deposition surface is critical to the process. Changes to the standoff distance change the workpiece illumination proportion (WIP) of the system, which describes the amounts of laser energy going directly into the substrate and wire [9]. Adjustment of the WIP is an important factor in the size and form of the laser spot. The effects of the WIP on process stability, bead geometry, substrate dilution, and mechanical properties have been studied by many research groups [9,10,11,12,13,14,15].

The coaxial deposition process is considered to be a directionally independent process by many users of the technology [7,16,17,18,19]. Its side-feed counterpart has been proven to be dependent on the travel direction with clear differences in a deposit that is front-fed and rear-fed [20]. The three-beam coaxial setup has a circularly asymmetric laser spot and, thus, must be investigated for potential directional effects. There is a gap in the current literature of directionality studies for the coaxial wire-delivery strategy, to either prove or disprove that the technology is directionally independent. Research related to the deposition direction in AM is commonly conducted on the infill pattern and layer orientation, such as the work conducted by Gabilondo et al. and Kurose et al. [21,22]. Though these analyses commonly find that the directional effect in AM is significant, this is a larger scale view of the directional effects. Starting the analysis of directionality at the scale of single deposition beads is important to observe the acute effect. The welding research community has studied these effects and generally finds that the welding direction can have an important effect on the resulting bead geometry and part distortion [23]. Veiga et al. studied bead symmetry in wire-arc AM and suggested an optimal welding technique to achieve the best bead symmetry [24,25]. These studies have not extended to the use of laser coaxial systems for additive manufacturing or laser welding. For various coaxial delivery strategies, lasers, feedstock materials, and process parameters, the confirmation of the directional independence for effects such as bead geometry, mechanical testing performance, and defect formation need to be further studied and outlined. The current paper studies the impact of deposition direction on the resulting bead shape and symmetry. Various symmetry measures are defined and utilized for the analysis of the process output. Using these measures, the process is evaluated for potential bead asymmetry with respect to the travel direction.

## 2. Materials and Methods

### 2.1. Experimental Setup

**Figure 1:**
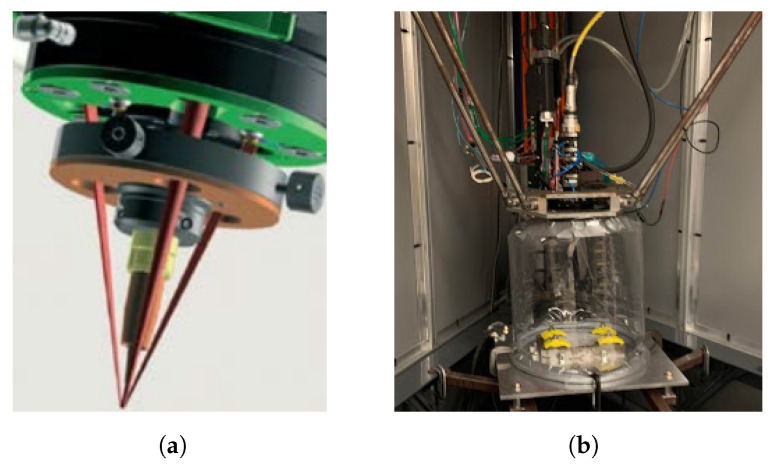
Setup of processing optics integrated with delta robot, laser input, and wire input [26]. (**a**) Fraunhofer COAXwire laser wire processing optics, (**b**) The processing optics is connected to the carriage of the delta robot.

The setup includes a laser, a wire-feeding system, a three-beam coaxial processing head, and a robot-positioning system. For specifics regarding the equipment used for this research, see the previous research paper from Mathenia et al., which used the same experimental setup [15]. The wire is a 1.2 mm diameter Ti-6Al-4V (Ti64), and the substrate surface is 6 mm thick Ti64 plates. Figure 1a shows the arrangement of the three laser beams and the wire [26]. Figure 1b shows the deposition cell. In support of the experimental research at Missouri University of Science and Technology (MST), GKN Aerospace provided the laser, coaxial wire deposition head, and titanium wire and substrates, as well as relevant technical support for the deposition cell.

**Figure 2:**
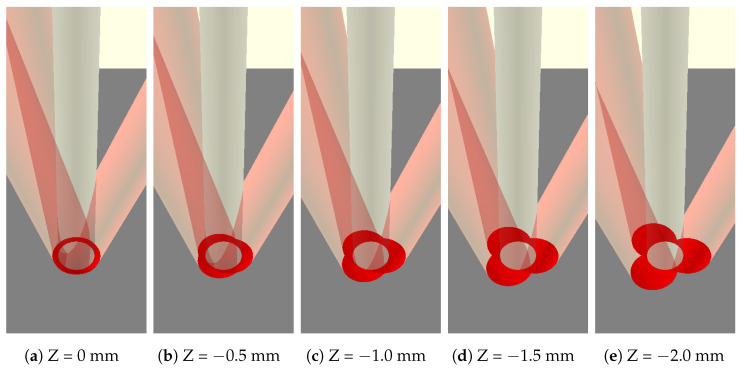
Beam spot patterns at various work plane offsets. The Z position denotes the distance between the beam convergence and work planes. WIP increases with increasing defocusing distance.

The standoff distance of the deposition head to the printing surface can be adjusted to alter the distribution of laser energy on the printed material. The WIP metric quantitatively describes this idea, specifying the proportion of laser energy that is delivered directly to the workpiece or substrate. The remainder of the laser energy is delivered directly to the wire. This is made possible by the laser delivery strategy of the Fraunhofer coaxial deposition head. Figure 2 shows multiple different standoff distance adjustments and the resulting beam spot pattern on the wire and substrate. In this study, the standoff distance and WIP will be held at a constant value.

### 2.2. Experimental Design

The experiment is intended to measure the impact or significance of the deposition direction on the bead profile symmetry of deposited tracks. Single bead tracks were deposited in various directions with respect to the three-laser-beam arrangement. The traverse feed rate and wire feed speed (WFS) were varied over three levels to measure if the deposition rate has an impact on the directional effects. The ratio of the traverse feed and wire feed speeds were held constant between the treatment combinations. Hereafter, the traverse feed rate will be referred to as the feed, and changes to the traverse feed rate will imply a proportional change to the WFS and deposition rate. Equation (1) shows the experimentally derived relationship between the process parameters. This relationship was found to result in a stable deposition by providing a constant amount of energy per unit length (J/mm) input to the feedstock.

(1) $$\frac{\mathrm{LP}\times (1-\mathrm{WIP})}{\mathrm{Feed}}=\mathrm{Constant}$$

Three parameter sets were deposited, as recorded in Table 1 below. The three parameter sets had increasing deposition rates. The second and third treatment combinations were 20% and 40% increases in the feed, WFS, and laser power (LP) from the first parameter set. Since the traverse feed rate and the wire feed speed were scaled proportionally, all three parameter sets had the same feed ratio and, thus, the same amount of input wire material per unit length of travel. The deposition rate will be determined as a significant or insignificant factor for directionality in coaxial printing.

**Figure 3:**
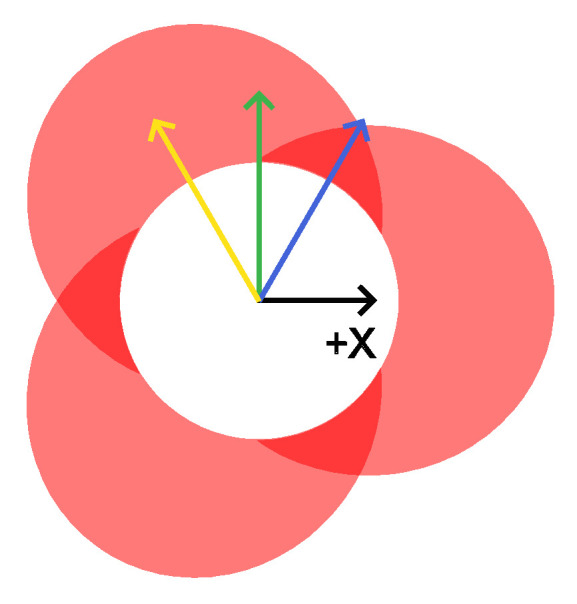
Laser spot pattern at Z = −1.29 mm produces WIP = 55%. The yellow, green, and blue lines represent the deposition travel directions. The yellow and blue directions are symmetric in the travel direction and represent traveling directly toward a beam and traveling directly between two beams, respectively. The green direction represents an asymmetric case where there are two laser beams on the left side and one laser beam on the right side of the travel direction.

**Figure 4:**
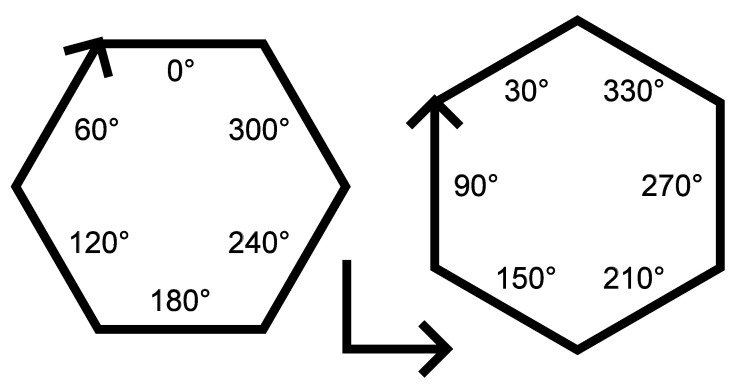
Planned deposition path to capture many different deposition directions. By printing two hexagons in this arrangement, twelve distinct directions can be easily deposited. The directions of each line are expressed in degrees from the positive *x*-axis. The L-shaped tracks at the bottom are aligned with the *x*- and *y*-axes of the machine and are used to determine the orientation during the analysis.

**Figure 5:**
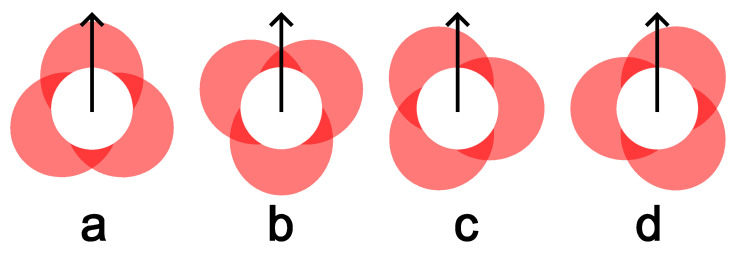
Various deposition directions with respect to the varying beam patterns. (**a**) Directly towards a beam, (**b**) Directly between two beams, (**c**) Left-skewed laser distribution, and (**d**) Right-skewed laser distribution.

For this study, the WIP value was held constant at 55%. The laser spot pattern for this standoff is shown in Figure 3. With the three beams spaced evenly around the wire, the laser beam arrangement relation to the feeding direction was variable. For instance, the deposit can be in a direction that is directly towards a laser beam, directly between two laser beams, or at any angle between them that skews the laser beam distribution to the left or the right of the travel direction. Figure 3 gives the direction vectors that illustrate this concept. To capture many different deposition directions, two hexagons were deposited for each treatment combination (TC) as arranged in Figure 4. This strategy provided twelve deposition directions, which were separated by 30 degrees. The twelve directions were broken down into the following four categories with three directions each: (a) traveling directly towards a laser beam (symmetric case about the travel direction), (b) traveling directly between two laser beams (symmetric), (c) left-skewed laser distribution about the travel direction (asymmetric), and (d) right-skewed laser distribution about the travel direction (asymmetric). Figure 5 shows the beam patterns with respect to the travel direction for each of these cases. With the deposits of the twelve travel directions, the beads were ready to be measured and analyzed to determine any differences between the deposition directions.

### 2.3. Experimental Procedure

**Figure 6:**
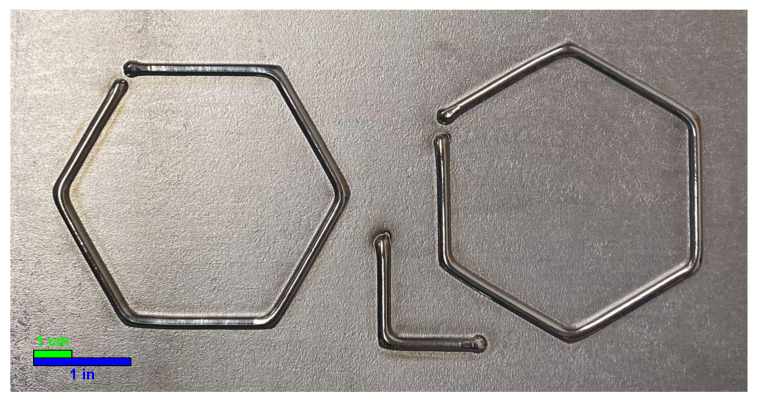
Sample deposited beads in the hexagonal pattern.

**Figure 7:**
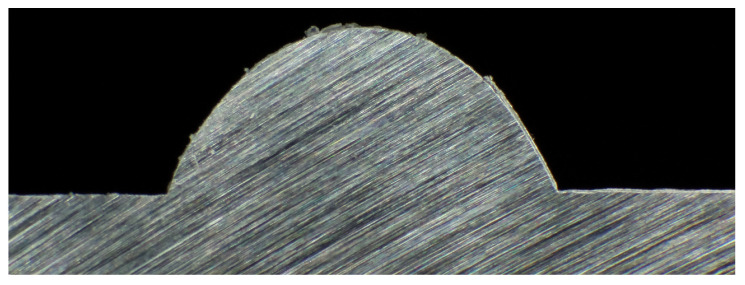
Sample cross-section of deposited bead.

**Figure 8:**
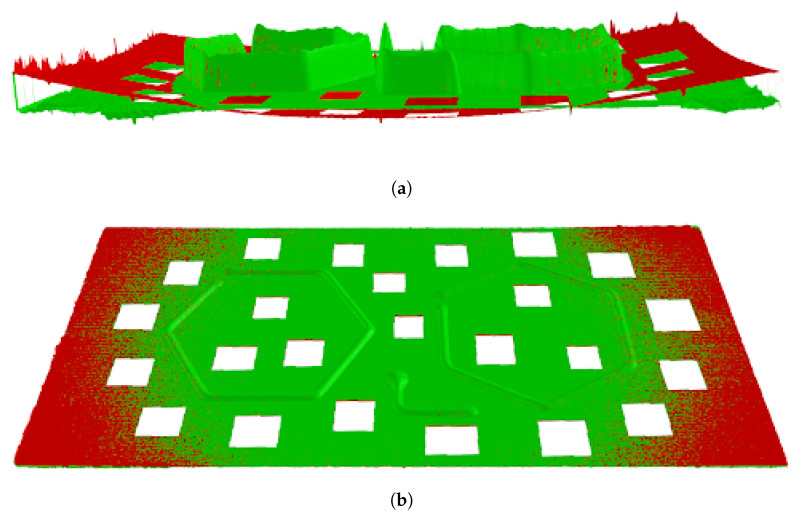
Sample deposits with the original scanned plate shown in red and the plate after flattening in green. This allows only the effects of the deposition to be measured and does not include the warpage induced by shrinkage. (**a**) Sample scanned plate with the bead height direction scaled by ten times to show the plate warpage; (**b**) sample scanned plate with the raw data overlaid onto the flattened deposition substrate.

**Figure 9:**
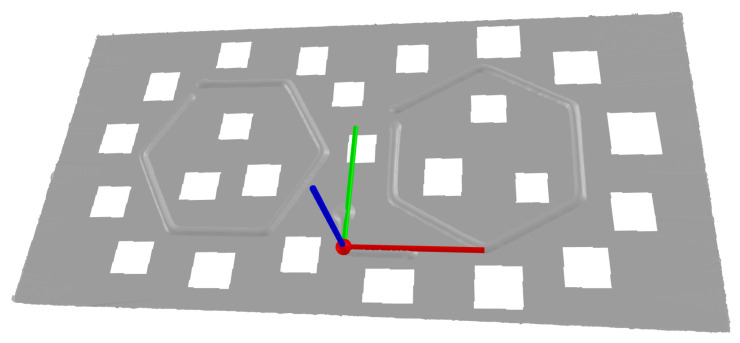
Alignment of the coordinate system of the scanned plate to a deposited feature in the positive X and Y directions. This alignment allows us to determine the location of the hexagons more easily.

**Figure 10:**
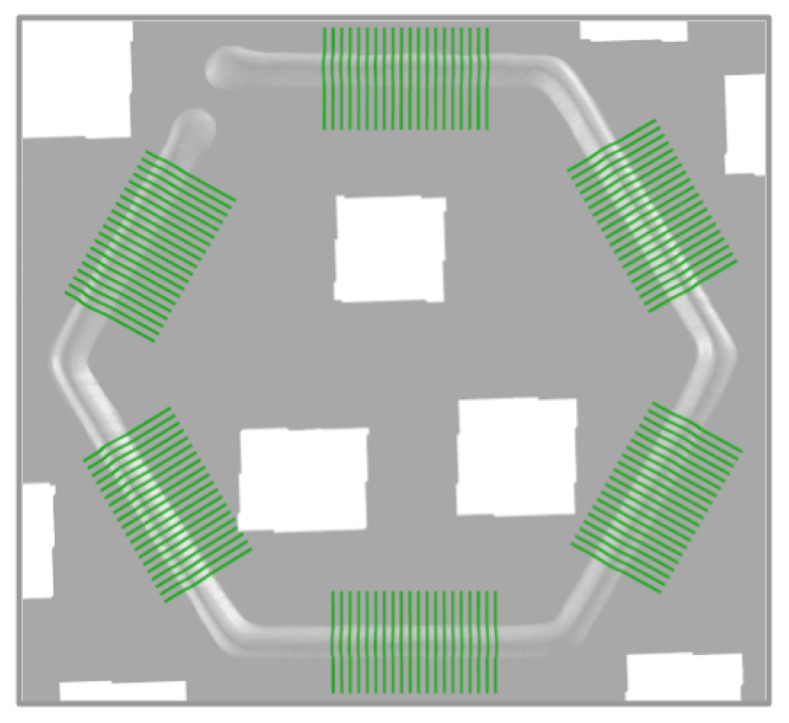
Slices taken from each of the sides of the hexagon to measure the bead profile of the deposit.

On each plate, two hexagons were printed according to the path shown in Figure 4 and the treatment combinations. The side length of each hexagon was 38.1 mm (1.5 in). A picture of a sample plate is shown in Figure 6 and Figure 7 shows a cross-section of a sample bead. A Revopoint MINI 3D Scanner (Revopoint, Shenzhen, China) was used to scan each plate. The scanner has a precision of 0.02 mm. A layer of AESUB Orange Scanning Spray was applied to the deposits to make the surface non-reflective and improve scannability. The layer thickness of the scanning spray is 2–6 µm. Scanning marker stickers were put onto the substrate surface to help with scanning accuracy and repeatability. The scan produced an STL file of the deposited beads and the substrate surface. The stickers were removed from the final STL file by cutting rectangles out of the locations with stickers as to not include them in the final analysis. These rectangles are shown in Figure 8, Figure 9 and Figure 10.

The original scan of the deposits shows that the substrate is bowed after deposition. Residual stresses that are induced during the heating and cooling processes cause the curvature in the plate. Figure 8 shows the raw scanned plate in red. To properly measure the effects of only the bead geometry, the curvature of the substrate must be removed. Points on the surface of the warped plate were used to create a C1 continuous interpolated surface. The reported bead profiles represent the differential height between the bead surface and the interpolated post-print plate surface. Figure 8 shows the flattened scanned plate in green. Next, to properly allow for locating the hexagon deposits, the coordinate system of the scan was moved and aligned with the deposited locating feature as pictured in Figure 9.

To locate each leg of the deposited hexagons, the known deposition-path-plan information was fed into the measurement program. Twenty cross-sectional slices centered on the midpoint of each deposited bead were taken for the analysis of the bead profile for each of the deposition directions. Figure 10 shows the slices on one of the deposited hexagons.

**Figure 11:**
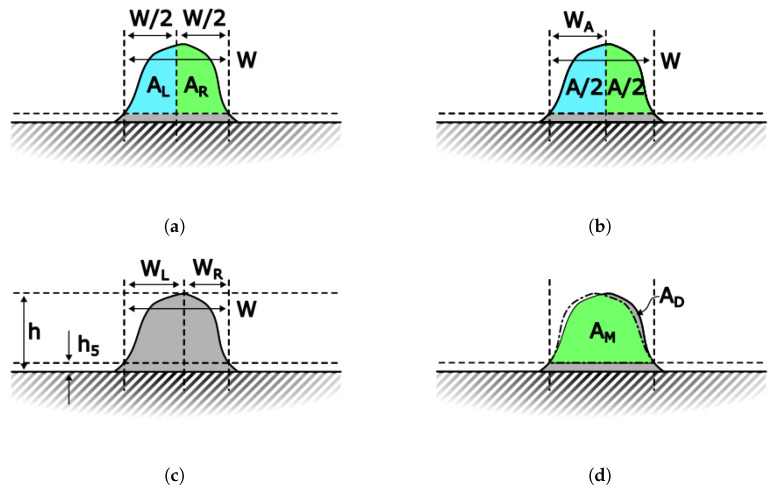
Bead measurement schematics and variable definitions. (**a**) Skew measurement schematic. We compare the areas to the left and the right of the half bead width to obtain a measure of the area distribution symmetry. (**b**) Lean measurement schematic. The bead width is compared to the position of the vertical line that cuts the bead into two equal areas. (**c**) Peak measurement schematic. The position of the maximum height of the bead is compared to the bead width. (**d**) Mirror measurement schematic. The bead is mirrored over the half bead width line, and the resulting overlapped area is compared to the original bead area.

With the cross-sectional bead profiles gathered for each of the deposition directions, quantitative measures of the bead asymmetry need to be defined and calculated. Four measures, named Skew, Lean, Peak, and Mirror, were developed to provide candidate methods to capture the asymmetries. Skew compares the areas of the left and right sides of the profile to measure the area distribution of the bead. Lean finds the vertical line that slices the bead into two equal areas. The position of this line is compared to the bead width to measure how centered this line is. Peak compares the location of the maximum along the profile to the total width to measure how centered the bead peak is. Mirror takes the bead profile and mirrors it over the half-bead-width vertical line. The overlapped area of the original bead and mirrored bead was compared to the area of the initial bead. To make all measures comparable and more comprehensible, each of the calculated values were remapped from 0 to 1 with 0 representing extreme asymmetry and 1 representing perfect symmetry. It should be noted that these remapped values do not capture whether the bead leans to the left or to the right. Figure 11 graphically shows each of the measures, and Equations (2)–(5) define the calculation and remapping of the measures.

(2) $$\mathrm{Skew}=1\;-\;|\frac{A_{L}\;-\;A_{R}}{A_{L}\;+\;A_{R}}|$$

(3) $$\mathrm{Lean}=1-2\;\times \;|\frac{W_{A}}{W}\;-\;0.5|$$

(4) $$\mathrm{Peak}=1-2\;\times \;|\frac{W_{L}}{W}\;-\;0.5|$$

(5) $$\mathrm{Mirror}=\frac{A_{M}}{A_{M}+A_{D}}$$

## 3. Results

**Figure 12:**
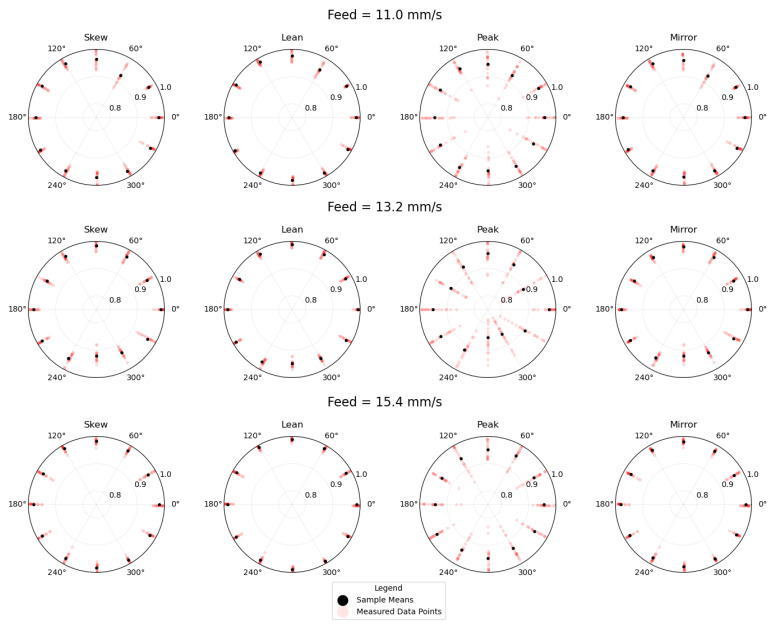
Polar scatter plots of Skew, Lean, Peak, and Mirror calculated values and sample means for the three deposited feed rates. The angular axis represents the absolute travel direction with respect to the machine positive x-axis, and the radial axis represents the specified calculated value.

The Skew, Lean, Peak, and Mirror metrics were measured and averaged at 20 positions along the length of each bead. Table 2 shows the absolute deposition direction with respect to the positive x-axis, the relative angle from the nearest travel direction that is directly towards a laser beam, the feed rate, and the four output measurements for all beads. Figure 12 shows the measured shows the data points and calculated sample means for each of the feed rates and each of the Skew, Lean, Peak, and Mirror metrics. The angular axis of each plot represents the absolute travel direction with respect to the positive x-axis, and the radial axis represents the calculated measured value.

## 4. Discussion

### 4.1. Experimental Discussion

To analyze the data in Table 2, an analysis of variance was conducted to determine if the tested treatment combinations had different sample means for the measured values of Skew, Lean, Peak, and Mirror. The statistical software JMP was used to carry out the analysis of variance. The models that were run were pairwise combinations of either the Absolute Angle or Relative Angle with Feed, and the response variable was Skew, Lean, Peak, or Mirror. This made for eight total models, which are shown in Table 3 with their respective *p*-values. Each analysis of variance model had 3 model degrees of freedom and 32 error degrees of freedom. At a 0.05 significance level, we failed to reject the null hypothesis that there is no difference between the treatment combination means.

The travel direction variable was broken into the measures of Absolute Angle and Relative Angle to capture two potential mechanisms of asymmetry in the deposition setup. The Absolute Angle travel direction could encode inconsistencies in the setup like a tilted deposition head or substrate, an uneven power distribution between the three lasers, or a wire input that is not centered between the lasers. From the first four analysis of variance models, we can see that the *p*-values are quite high and suggest that there are no such inconsistencies in the deposition setup when measuring bead symmetry as the output.

The Relative Angle encodes the angle between the travel direction and the nearest direction that is directly towards a laser beam. A Relative Angle of 0° represents a travel direction that is directly towards a laser beam (Figure 5a); 60° represents a travel direction that is directly between two beams (Figure 5b); 30° represents the asymmetric cases that have a left- or right-skewed laser distribution (Figure 5c,d). For each of the models that were run with the Relative Angle, the *p*-Values were lower for the Skew, Lean, Peak, and Mirror responses, but were all still insignificant at a 0.05 significance level. We again failed to reject the null hypothesis and concluded that the symmetry of the deposited beads is not dependent on travel direction with respect to the laser spot orientation over the tested parameter ranges.

### 4.2. Experiment Validation

**Figure 13:**
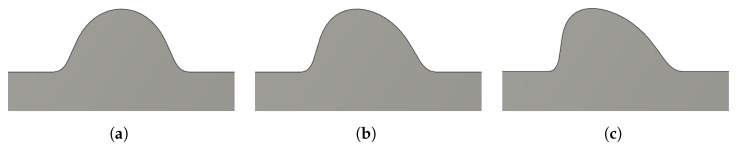
Bead profiles of varying amounts of asymmetry used for validation of the experimental process, equipment, and measurement tools. The bead profiles were generated using Fusion 360. (**a**) Nominal profile. The bead profile is perfectly symmetric. (**b**) Minor bead skew. The bead profile leans slightly to the left. (**c**) Major bead skew. The bead profile is severely skewed left.

With the analysis of variance showing that the treatment combination means were equal for all models, we felt that the validation of the experimental process and tools was necessary. If the process of depositing a bead, scanning it with a 3D scanner, and measuring it through the Skew, Lean, Peak, and Mirror measures could not detect bead profile asymmetries, then the above experiment would be useless. To test the process, three bead profiles were generated using a computer-aided design software that had varying amounts of asymmetry, as shown in Figure 13. To generate these profiles, the peaks of the beads were set at 50%, 40%, and 30% along the bead width, respectively. These beads were converted to STL objects and printed using an AnyCubic Photon D2 resin printer. The scale of these beads was similar to that of the titanium beads deposited for the experiment.

The printed beads were subjected to the same 3D scanning process as described above. The beads were first sprayed to make the surface consistent, non-reflective, and able to be scanned by the Revopoint MINI 3D Scanner. The scanned objects were processed and measured as described in the Experimental Procedure. Many bead profiles were sampled from each of the beads, and the Skew, Lean, Peak, and Mirror measures were calculated for each cross-section. The calculated measures were averaged, and these averages are reported in Table 4.

From the values in the above table, a few observations can be made. From the Nominal bead to the Major bead, Peak had the largest range, while Lean has the tightest range. For all measures, the jump from the Nominal to Minor case was markedly larger than the jump from the Minor to Major case. For all measures, there seemed to be a clear and obvious difference between the measured symmetric bead and the asymmetric beads. Skew and Mirror yielded nearly identical values for each of the three beads. Both of these measures are area-based and appeared to quantitatively respond very similarly to changes in the bead shape.

**Figure 14:**
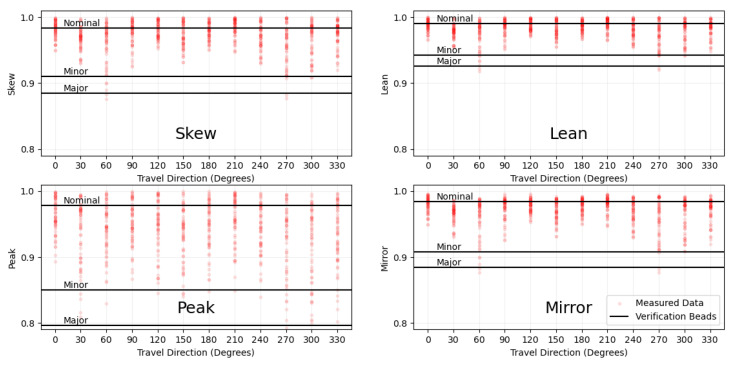
Plots showing experimentally measured data for Skew, Lean, Peak, and Mirror against travel direction in degrees from the positive x-axis, as well as the calculated mean value for the three validation beads.

To view the averages of the measured values for the validation beads against the experimental data, the plots in Figure 14 were made. The red dots represent the collected experimental data points over all travel directions and feed rates, and the black horizontal lines represent the averages for the validation beads shown in Table 4. It is immediately obvious that the Peak measurement technique had a considerably higher range and potential noise in the measurement. Since the scanned object is a triangulated STL object, we hypothesized that the Peak measurement was quite sensitive to the effect of the triangulation and the location of the cross-section along the bead. Due to these effects, we believe that the Peak measurement technique should not be used to measure bead asymmetries for 3D scanned beads. The Skew, Lean, and Mirror methods are all based on the area distribution of the cross-section of the bead, and all seemed to be more stable measures. For the 720 plotted points on each graph, most of the data were clustered around the Nominal symmetric bead with some outliers reaching the Minor and Major cases of asymmetry. Based on the clear differences between the Nominal case and the Minor case for the validation beads and this clustering around the Nominal case, it is fair to conclude that the deposition setup produced generally symmetric beads for all travel directions. Skew and Mirror had a noticeably larger range than Lean, but seemed to simply encode the same trends over the larger numerical range. These three measures, Skew, Lean, and Mirror, can all be used to consistently measure asymmetries in bead profiles. With additional validation experiments, more asymmetry cases can be tested, quantified, and classified for each of the measures.

## 5. Conclusions

This paper consists of an experimental analysis of bead geometry symmetry in coaxial wire-based laser metal deposition. Quantitative measurement methods were developed and used to determine the usefulness of the metrics and if the deposition setup had directional independence with respect to the bead geometry. The experimental process of deposition, the use of a 3D scanner, and data analysis were verified using intentionally asymmetric bead profiles. The research yields the following conclusions:

- Over the experimental settings, the three-beam coaxial wire-based laser metal deposition setup was found to have no directional effect on the symmetry of the output bead geometry. The system was directionally independent of the absolute travel direction and the travel direction relative to the laser spot distribution.
- The Skew, Lean, and Mirror measurements were found to effectively capture varying amounts of asymmetry in the profile of a deposited bead. All three measures were based on the bead area distribution and provided viable options to quantify bead profile symmetry.
- The Peak measurement was found to be noisy and inconsistent, likely due to the triangulation of the 3D scanned object. The Peak measure was not area-based and, instead, was based on the position of the maximum relative to the bead width. A very fine STL mesh could be used to alleviate this problem for the Peak measurement, at the slight expense of increased scan file sizes and data processing time.

Coaxial wire-based laser metal deposition can be trusted as a directionally independent process with respect to the bead geometry. The distinctive characteristics and capabilities of the coaxial technology need to be further studied to extend its use to practical, industrial, and commercial applications. Research into three-beam coaxial directionality can be extended to various deposition heads, feedstock materials, and process parameters to determine if the setup is truly directionally independent in most or all engineering uses.

## Figures and Tables

**Table 1:** Experimental process parameters.

Treatment Combination	Feed (mm/s)	WFS (mm/s)	WIP (%)	LP (W)
1	11.0	38.3	55.0%	1400
2	13.2	46.0	55.0%	1680
3	15.4	53.6	55.0%	1960

**Table 2:** Experimental data.

Absolute Angle (°)	Relative Angle (°)	Feed (mm/s)	Skew	Lean	Peak	Mirror
0	0	11.0	0.9775	0.9847	0.9574	0.9751
30	30	11.0	0.9718	0.9804	0.9637	0.9713
60	60	11.0	0.9272	0.9516	0.9291	0.9269
90	30	11.0	0.9635	0.9762	0.9451	0.9596
120	0	11.0	0.9789	0.9863	0.9572	0.9778
150	30	11.0	0.9801	0.9869	0.9521	0.9764
180	60	11.0	0.9732	0.9821	0.9456	0.9726
210	30	11.0	0.9891	0.9929	0.9524	0.9857
240	0	11.0	0.9770	0.9851	0.9615	0.9754
270	30	11.0	0.9699	0.9806	0.9452	0.9687
300	60	11.0	0.9781	0.9860	0.9575	0.9771
330	30	11.0	0.9771	0.9848	0.9417	0.9763
0	0	13.2	0.9875	0.9918	0.9755	0.9861
30	30	13.2	0.9628	0.9754	0.8997	0.9611
60	60	13.2	0.9734	0.9828	0.9411	0.9719
90	30	13.2	0.9840	0.9898	0.9559	0.9799
120	0	13.2	0.9754	0.9849	0.9305	0.9723
150	30	13.2	0.9605	0.9744	0.9060	0.9591
180	60	13.2	0.9814	0.9876	0.9511	0.9785
210	30	13.2	0.9813	0.9874	0.9490	0.9762
240	0	13.2	0.9561	0.9717	0.9220	0.9544
270	30	13.2	0.9202	0.9487	0.8532	0.9201
300	60	13.2	0.9336	0.9581	0.8543	0.9330
330	30	13.2	0.9669	0.9784	0.9072	0.9622
0	0	15.4	0.9799	0.9868	0.9559	0.9786
30	30	15.4	0.9674	0.9787	0.9458	0.9645
60	60	15.4	0.9795	0.9867	0.9558	0.9756
90	30	15.4	0.9822	0.9885	0.9512	0.9814
120	0	15.4	0.9873	0.9919	0.9462	0.9819
150	30	15.4	0.9770	0.9850	0.9453	0.9730
180	60	15.4	0.9815	0.9876	0.9441	0.9797
210	30	15.4	0.9806	0.9871	0.9665	0.9782
240	0	15.4	0.9772	0.9854	0.9431	0.9751
270	30	15.4	0.9819	0.9887	0.9471	0.9795
300	60	15.4	0.9856	0.9910	0.9346	0.9809
330	30	15.4	0.9762	0.9844	0.9467	0.9742

**Table 3:** Analysis of variance *p*-values of various models.

Factors	Response	*p*-Value
Absolute and Feed	Skew	0.5414
Absolute and Feed	Lean	0.5114
Absolute and Feed	Peak	0.8331
Absolute and Feed	Mirror	0.5702
Relative and Feed	Skew	0.2641
Relative and Feed	Lean	0.2887
Relative and Feed	Peak	0.7272
Relative and Feed	Mirror	0.3085

**Table 4:** Quantitative symmetry measures for experiment validation beads.

Bead	Skew	Lean	Peak	Mirror
Nominal	0.9840	0.9902	0.9781	0.9842
Minor	0.9106	0.9428	0.8503	0.9082
Major	0.8851	0.9260	0.7958	0.8848

## Data Availability

The original contributions presented in the study are included in the article. Further inquiries can be directed to the corresponding author.

## Abbreviations

The following abbreviations are used in this manuscript:

AM	additive manufacturing
DED	directed energy deposition
LP	laser power
MST	Missouri University of Science and Technology
Ti64	Ti-6Al-4V
TC	treatment combination
WFS	wire feed speed
WIP	workpiece illumination proportion
